# Uncovering the Mechanisms of Halotolerance in the Extremely Acidophilic Members of the *Acidihalobacter* Genus Through Comparative Genome Analysis

**DOI:** 10.3389/fmicb.2019.00155

**Published:** 2019-02-08

**Authors:** Himel N. Khaleque, Carolina González, Raihan Shafique, Anna H. Kaksonen, David S. Holmes, Elizabeth L. J. Watkin

**Affiliations:** ^1^School of Pharmacy and Biomedical Sciences, Curtin Health Innovation Research Institute, Curtin University, Perth, WA, Australia; ^2^CSIRO Land and Water, Floreat, WA, Australia; ^3^Center for Bioinformatics and Genome Biology, Science for Life Foundation, Santiago, Chile; ^4^Sodexo Australia, Perth, WA, Australia; ^5^Centro de Genómica y Bioinformática, Facultad de Ciencias, Universidad Mayor, Santiago, Chile

**Keywords:** acidophile, halophile, *Acidihalobacter*, osmoprotectant, ectoine, astrobiology

## Abstract

There are few naturally occurring environments where both acid and salinity stress exist together, consequently, there has been little evolutionary pressure for microorganisms to develop systems that enable them to deal with both stresses simultaneously. Members of the genus *Acidihalobacter* are iron- and sulfur-oxidizing, halotolerant acidophiles that have developed the ability to tolerate acid and saline stress and, therefore, have the potential to bioleach ores with brackish or saline process waters under acidic conditions. The genus consists of four members, *A. prosperus* DSM 5130^T^, *A. prosperus* DSM 14174, *A. prosperus* F5 and “*A. ferrooxidans*” DSM 14175. An in depth genome comparison was undertaken in order to provide a more comprehensive description of the mechanisms of halotolerance used by the different members of this genus. Pangenome analysis identified 29, 3 and 9 protein families related to halotolerance in the core, dispensable and unique genomes, respectively. The genes for halotolerance showed K_a_/K_s_ ratios between 0 and 0.2, confirming that they are conserved and stabilized. All the *Acidihalobacter* genomes contained similar genes for the synthesis and transport of ectoine, which was recently found to be the dominant osmoprotectant in *A. prosperus* DSM 14174 and *A. prosperus* DSM 5130^T^. Similarities also existed in genes encoding low affinity potassium pumps, however, *A. prosperus* DSM 14174 was also found to contain genes encoding high affinity potassium pumps. Furthermore, only *A. prosperus* DSM 5130^T^ and “*A. ferrooxidans*” DSM 14175 contained genes allowing the uptake of taurine as an osmoprotectant. Variations were also seen in genes encoding proteins involved in the synthesis and/or transport of periplasmic glucans, sucrose, proline, and glycine betaine. This suggests that versatility exists in the *Acidihalobacter* genus in terms of the mechanisms they can use for halotolerance. This information is useful for developing hypotheses for the search for life on exoplanets and moons.

## Introduction

The most extensively studied acidophiles are those that oxidize iron and/or sulfur for the catalytic dissolution of minerals in low pH environments (Johnson and Schippers, 2017). These microorganisms can be used in biomining, i.e., for the economic extraction of metals from low grade ores, which are otherwise too expensive to process through traditional mining processes such as smelting or roasting (Rohwerder et al., 2003).

In regions like Western Australia and Chile, groundwater is often limited, and seawater may be used for bioleaching operations. Moreover, with decreasing ore grades, mining companies are moving toward the use of low-grade, more complex ores, which may contain impurities, such as chloride. High concentration of chloride in process waters inhibits the growth of acidophiles, decreasing the bioleaching rates and yields. Desalination can be used to remove chloride ion but this is prohibitively expensive (Petry et al., 2007; Watling, 2016; Zammit and Watkin, 2016). While anions such as sulfate and cations such as sodium ions limit the growth of acidophiles due to their ability to cause osmotic stress, the biggest challenge to biomining operations using seawater is caused by chloride ions. These ions can cross the cell membrane and cause acidification of the cytoplasm by disrupting the reverse transmembrane potential and thereby inhibiting cell growth and ultimately causing cell death (Blight and Ralph, 2004; Shiers et al., 2005; Davis-Belmar et al., 2008; Rea et al., 2015; Boxall et al., 2016). Therefore, the discovery and characterization of halophilic acidophiles that can tolerate chloride ion concentrations greater than the 19 g/L present in seawater is important to the mining industry as they offer a means of leaching base metals with saline water and from high-salt ores (Zammit et al., 2009; Watling, 2016).

Most studies on the halotolerance of microorganisms at low pH have been conducted on pathogens that only have brief exposure to acid stress (Zammit and Watkin, 2016). Likewise, most of the studies on extreme halophiles have been undertaken under neutral or alkaline conditions (Empadinhas and da Costa, 2008). The combination of high salinity and low pH drastically reduces the number of organisms which can survive in this ecological niche. Despite the ongoing search for halotolerant acidophiles over the past 20 years, only a few microorganisms that are capable of oxidizing iron and sulfur in the presence of salt and acid stress simultaneously have been identified (Watling, 2016; Zammit and Watkin, 2016). This is because there are only a few geographical locations where both low pH and high salt environments exist, such as acidic saline lakes and drains and volcanoes near seawater (Zammit and Watkin, 2016). Members of the acidophilic and halotolerant species of the *Acidihalobacter* genus represent a group of Gram-negative, halophilic, iron- and sulfur-oxidizing, mesophilic, chemolithoautotrophic, extreme acidophiles that have been isolated from these unique environments (Huber and Stetter, 1989; Simmons and Norris, 2002; Zammit et al., 2009). To date, only four members of the *Acidihalobacter* genus have been characterized. The first member of the genus to be identified was *A. prosperus* DSM 5130^T^, isolated from a geothermal heated seafloor at Porto di Levante, Vulcano, Italy and shown to have a chloride ion tolerance of 35 g/L (Huber and Stetter, 1989). More recently, *A. prosperus* DSM 14174 and “*A. ferrooxidans*” DSM 14175 were isolated from hydrothermal pools at the Aeolian Islands, Vulcano, Italy (Simmons and Norris, 2002). Another isolate, *A. prosperus* F5 was the first of this species to be isolated in Australia from a mixed environmental culture obtained from an acidic saline drain (Zammit et al., 2009). All three of these isolates were found to tolerate up to 45 g/L chloride ion and to leach base metals from pyrite at up to 30 g/L chloride ion (Khaleque et al., 2017a,b). Furthermore, *A. prosperus* DSM 14174 was able to leach copper from a copper containing ore at 30 g/L chloride ion in the presence of “*A. ferrooxidans*” DSM 14175 and other salt-tolerant acidophiles (Davis-Belmar et al., 2008). Additionally, a pure culture of *A. prosperus* F5 could leach chalcopyrite at 18 g/L chloride ion and pentlandite at 45 g/L chloride ion (Khaleque et al., 2017a). The ability of these microorganisms to release metals from insoluble ores in the presence of acid and salt stress make them worthy candidates for elucidation of the mechanisms of salt stress tolerance in acidophiles.

Genome sequencing can be an important first step in characterizing a new organism as it provides critical genetic information required to elucidate biochemical pathways underpinning its metabolic capabilities and survival mechanisms. Several acidophiles have been sequenced and comparative genomics has shed light on their metabolic processes (Tyson et al., 2004; Levicán et al., 2008; Valdés et al., 2008, 2010; Zhou et al., 2008; Cárdenas et al., 2010, 2012; Zhang et al., 2016a,b; Tran et al., 2017). The sequencing of the genomes of the members of the *Acidihalobacter* genus (Ossandon et al., 2014; Khaleque et al., 2017a,b,c) has provided an opportunity to better study the mechanisms of survival used by these acidophilic, halotolerant acidophiles.

In this study, comparative genomic analysis of all members of the *Acidihalobacter* genus was used to enhance the understanding of the mechanisms these acidophiles employ to tolerate salt stress.

## Materials and Methods

### *Acidihalobacter* Genome Sequencing, Annotation and Comparisons

Genome sequencing and assembly were performed as previously described (Ossandon et al., 2014; Khaleque et al., 2017a,b,c). Genome sequences have previously been deposited at DDBJ/ENA/GenBank with the following accession numbers: *A. prosperus* DSM 5130^T^ (JQSG00000000.2), *A. prosperus* F5 (CP017415.1), *A. prosperus* DSM 14174 (CP017448.1) and “*A. ferrooxidans*” DSM 14175 (CP019434.1).

The genome sequences were annotated using Rapid Annotation using Subsystem Technology (RAST) server^1^ using the ClassicRAST annotation scheme (Aziz et al., 2008; Overbeek et al., 2014). Metabolic pathways were predicted by Kyoto Encyclopedia of Genes and Genomes (KEGG)^2^. Whole genomes were aligned and compared by the construction of a circular genomic map for each genome using the BLAST Ring Image Generator (BRIG, version 0.95) as described by Alikhan et al. (2011). The complete genome sequence of *A. prosperus* F5 was used as the reference sequence for the *Acidihalobacter* genus. The genome maps were drawn on a local BLAST basis using an *E*-value of 1e-5 and upper and lower identity threshold percentages of 70% and 50%, respectively.

Predicted protein sequences corresponding to all *Acidihalobacter* proteomes were sorted using an all-vs-all BLASTP script based on Best Bidirectional BLAST Hit (BBBH; Altschul et al., 1997) with an *E*-value cut off of 1e-5. Protein families were constructed based on 50% of identity and coverage of alignments, assigning each protein to one protein family (Snipen and Ussery, 2010). The protein families were classified in core-, dispensable- and unique-genome.

### Identification of Genes Involved in Halotolerance in *Acidihalobacter*

Potential mechanisms of halotolerance were identified through an extensive literature search for mechanisms related to halotolerance that have previously been identified in other microorganisms. These genes were then searched in *Acidihalobacter* species genomes through manual curation and by BLAST comparisons (minimal *E*-value of 1e-5) using Geneious v.8.1.8 bioinformatics software (Kearse et al., 2012; Overbeek et al., 2014). Synteny blocks between *Acidihalobacter* genomes and conservation of gene neighbors were determined by MAUVE (Darling et al., 2010). Genomic contexts were visualized using Artemis (Rutherford et al., 2000), the RAST server (Aziz et al., 2008) and Geneious v. 8.1.8 software (Kearse et al., 2012; Overbeek et al., 2014).

Amino acid alignments of families related to halotolerance were aligned using MUSCLE (Edgar, 2004). The alignments were used as input for PAL2NAL (Suyama et al., 2006) in conjunction with their nucleotide sequences to obtain their codon alignments. The ratio of non-synonymous (K_a_) to synonymous (K_s_) nucleotide substitution rates (K_a_/K_s_ ratios) were calculated using SeqinR package of R project (Charif and Lobry, 2007). Mean K_a_/K_s_ ratios were assigned for halotolerant resistance families, with ratios of > 1 indicating beneficial mutations and ratios of < 1 indicating purifying selection (Li et al., 1985).

## Results and Discussion

In general, acidophiles maintain a near neutral intracellular pH despite a proton gradient of up to 10,000 fold across the cytoplasmic membrane (Baker-Austin and Dopson, 2007). This is achieved through a variety of mechanisms including: the maintenance of a positive membrane potential to reduce proton influx by electrostatic repulsion (through the accumulation of K^+^); using active proton pumps to export protons; altering their cytoplasmic membrane structures; the use of enzymes such as carboxylases to consume protons; and through various cytoplasmic buffering systems (Baker-Austin and Dopson, 2007). Furthermore, the Omp40 protein in *Acidithiobacillus ferrooxidans* has also previously been shown to be a small, slightly anionic pore hypothesized to prevent the movement of protons across its outer membrane (Guiliani and Jerez, 2000). Sodium chloride has a deleterious effect on acidophiles due to its ability to cause osmotic stress. Chloride, however, is far more harmful to the cells due to the ability of chloride ions to permeate the cell membrane leading protons to be electrostatically attracted to the increasing negative charge in the cell (Alexander et al., 1987; Grogan, 1989; Suzuki et al., 1999; Zammit et al., 2009, 2012). This results in acidification of the cytoplasm and a collapse of the reverse transmembrane potential, which ultimately results in cell death. While the effect of both osmotic stress and chloride on acidophilic microorganisms is well understood, mechanisms of halotolerance in acidophiles that can withstand high levels of salt, such as the members of the *Acidihalobacter* genus, are not well studied as only a few halotolerant, acidophilic iron and sulfur oxidizing microorganisms have been discovered. Therefore, analysis of the genomes of the members of this genus was undertaken to identify potential mechanisms of osmotic stress and chloride stress tolerance at low pH.

**FIGURE 1:**
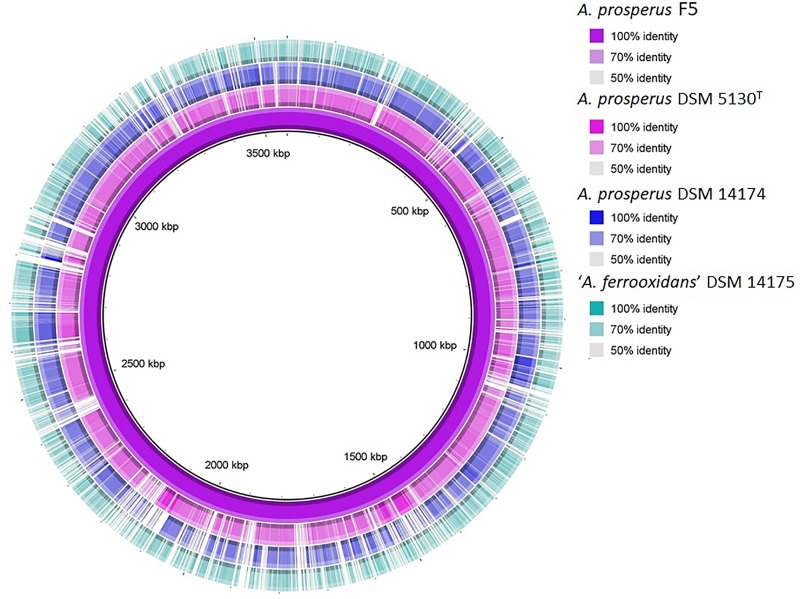
Whole genome comparisons of the members of the *Acidihalobacter* genus using the complete genome of *A. prosperus* F5 as the reference sequence. The BLAST match identity is represented by the color intensity in each ring.

The genus *Acidihalobacter* is represented by four acidophilic, iron and sulfur oxidizing mesophiles that demonstrate a chemolithoautotrophic lifestyle and are able to bioleach metals from insoluble ores in the presence of higher salt stress than have previously been reported for other acidophilic biomining microorganisms (Huber and Stetter, 1989; Simmons and Norris, 2002; Norris and Simmons, 2004; Davis-Belmar et al., 2008; Nicolle et al., 2009; Ossandon et al., 2014; Cárdenas et al., 2015; Khaleque et al., 2017a,b,c, 2018a). The genomes of the isolates of the *Acidihalobacter* genus have all recently been sequenced (Ossandon et al., 2014; Khaleque et al., 2017a,b,c). The genomic features of the members of the *Acidihalobacter* genus are compared in Table 1. All genomes ranged in size between 3.36 and 3.57 Mbp and a GC content of 59.9–64.5%. Based on 99% sequence identity of the 16s rRNA sequence of *A. prosperus* DSM 14174 and *A. prosperus* F5 to the type strain, *A. prosperus* DSM 5130 (as given by BLAST), the former two strains are classified as the *A. prosperus* species. However, “*A. ferrooxidans*” has only 97% 16s rRNA sequence identity to *A. prosperus* DSM 5130 and *A. prosperus* DSM 14174 and 96% identity to *A. prosperus* F5, and is therefore believed to be a new species of the *Acidihalobacter* genus (unpublished data). *A. prosperus* DSM 14174 was the only isolate found to contain a plasmid. *A. prosperus* F5 was the only genome for which a complete sequence, with no gaps, was obtained and was, therefore, used as the reference sequence for comparison and visualization of circular genomic maps using BRIG (Alikhan et al., 2011). The resulting genomic maps (Figure 1) highlighted the similarity in the genomes of the members of the *Acidihalobacter* genus, suggesting conservation of genes amongst the species.

**Table 1:** Comparison of the genomic features of the four members of the *Acidihalobacter* genus.

Feature	*A. prosperus* DSM 5130^T^	*A. prosperus* DSM 14174	*A. prosperus* F5	“*A. ferrooxidans*” DSM 14175
Genome size (Mbp)	3.36	3.36	3.57	3.45
GC content (mol%)	64.5	62.2	59.9	61.6
Coding DNA sequence (CDS)	3,088	3,194	3,233	3,089
Plasmid	Not present	162,484 bp	Not present	Not present
tRNA genes	48	46	47	45


**FIGURE 2:**
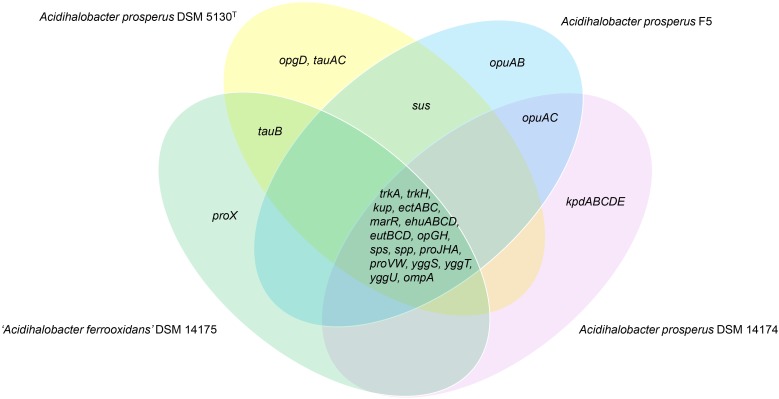
Venn diagram showing the shared and unique genes related to halotolerance in members of the *Acidihalobacter* genus.

**FIGURE 3:**
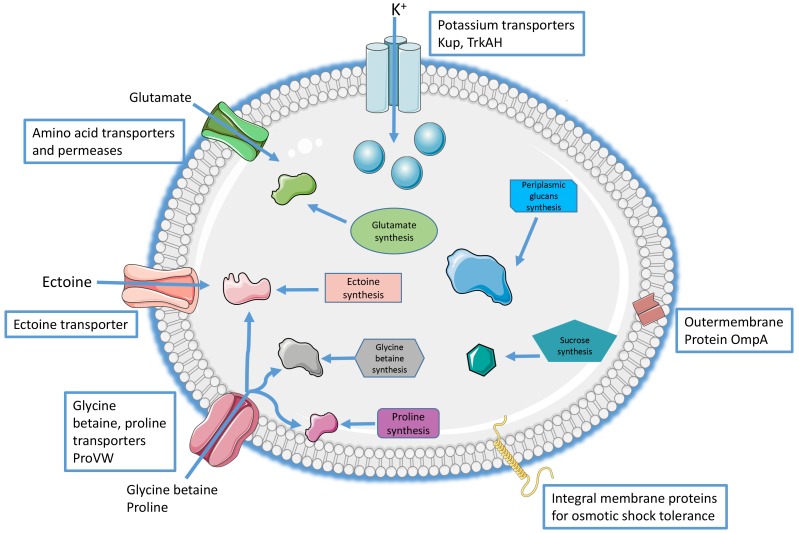
Potential mechanisms of halotolerance shared by the members of the *Acidihalobacter* genus.

Pangenome models are extremely useful in the characterization of the gene repertoire of microbes and as a tool to investigate the biology and lifestyle of individual microorganisms as well as the definition of species (Tettelin et al., 2008). Therefore, to investigate the differences between the genomes and to identify protein families that may play a role in the halotolerance of the members of the *Acidihalobacter* genus, pangenome analysis was performed. The complete gene pool of all *Acidihalobacter* species were found to consist a total of 6,243 protein families forming the pangenome. The pangenome was subsequently classified into the core-genome and the accessory genome (dispensable and unique genome). The core genome consists of 1422 protein families shared by all strains whereas the dispensable genome consists of 1496 protein families assigned to less than four but more than one strain. The unique genome consists of 3325 protein families assigned to only one strain. A total of 41 genes with roles in halotolerance were selected from the pangenome for further analysis. Of these, 29 were found in the core genome, 3 in the dispensable genome and 9 in the unique genome. Selected genes with potential roles in halotolerance in the members of the *Acidihalobacter* genus are shown in Table 2. Differences in the presence of genes for halotolerance between the species/strains are shown in Figure 2. Halotolerance mechanisms shared by the members of the *Acidihalobacter* genus are shown in Figure 3.

**Table 2:** Genes and their encoded proteins with potential roles in halotolerance in members of the *Acidihalobacter* genus.

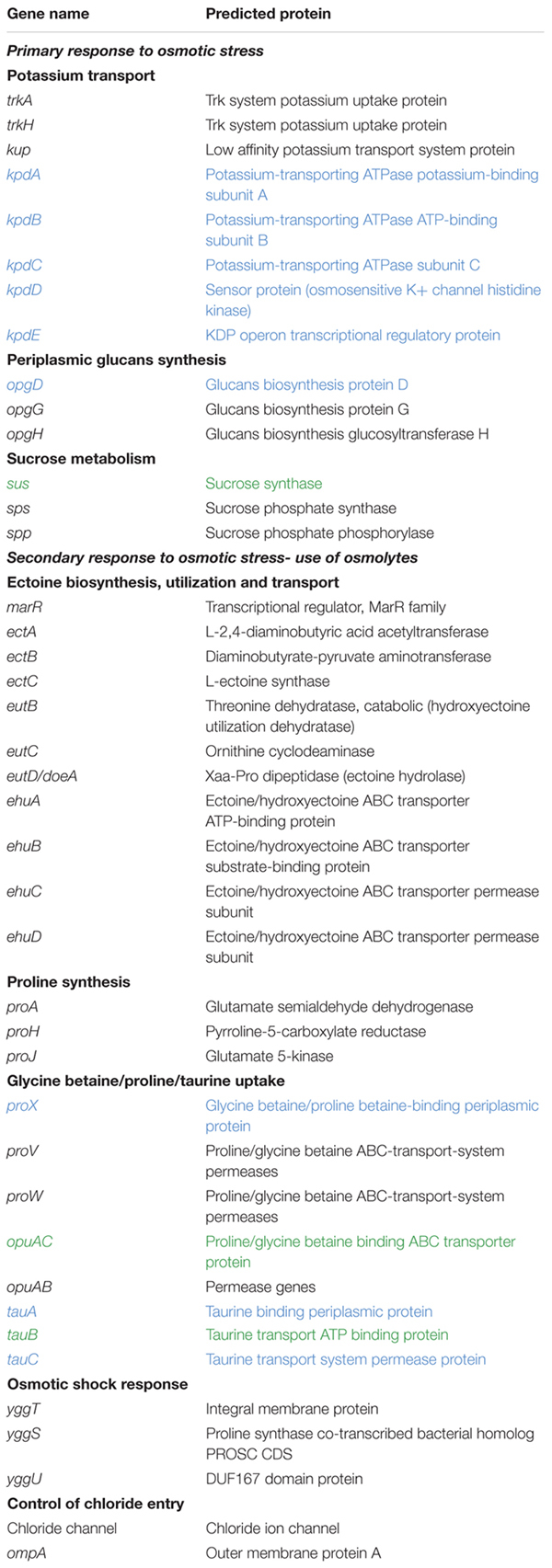

*Black text, Core genome; Green text, Dispensable genome; Blue text, Unique genome.*

**FIGURE 4:**
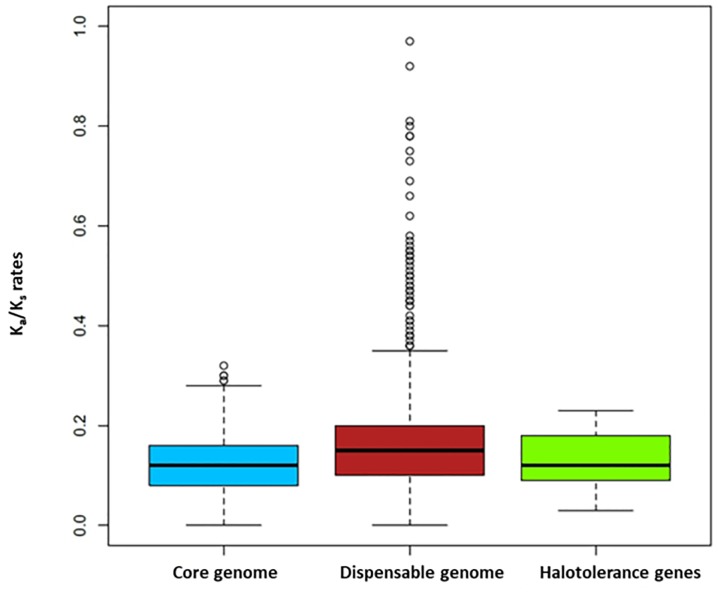
Boxplot of K_a_/K_s_ ratios of core-genome (blue), dispensable-genome (red) and halotolerance genes (green).

It has previously been suggested that the core genome consists of protein families shared by all strains that encode functions related to the basic biology and phenotypes of a species (Tettelin et al., 2008). The protein families in the core genome of the members of the *Acidihalobacter* genus have important roles for pathways related to energy acquisition, carbohydrate metabolism and adaptation to extreme environments. Likewise, the majority of genes related to halotolerance in *Acidihalobacter* species were found in the core-genomes. This is not surprising, as it is expected that *Acidihalobacter* species are able to survive their extreme environments through well-conserved mechanisms. In order to confirm this, calculation of K_a_/K_s_ ratios was performed in order to determine the magnitude and direction of natural selection on protein coding genes. For a given period, K_a_/K_s_ ratios calculate the number of non-synonymous substitutions per non-synonymous sites (K_a_) to the number of synonymous substitutions per synonymous site (K_s_) in order to determine the net balance between beneficial and non-beneficial mutations. Ratios of significantly more than 1 indicate that mutations that have occurred are beneficial whereas values of less than 1 indicate purifying selection (tendency to be stabilized and against change) (Li et al., 1985). For the core genome of the members of the *Acidihalobacter* genus, K_a_/K_s_ ratios were found to be between 0 and 0.2, suggesting that the protein families forming the core genome are well conserved. Similarly, the genes involved in halotolerance also showed ratios between 0 and 0.2 (Figure 4). This confirmed that there is pressure to conserve the sequences of housekeeping genes in order to maintain stable sequences that allow *Acidihalobacter* species to survive salt stress conditions.

Closer inspection of the mechanisms of halotolerance used by the members of the *Acidihalobacter* genus was undertaken by investigation of the possible roles of the genes involved in the primary and secondary responses to osmotic stress as well as those with roles in chloride tolerance, as described below.

### Primary Responses of *Acidihalobacter* spp. to Osmotic Stress

The genomes of all described species of the *Acidihalobacter* genus harbor 2–3 copies of genes encoding energy dependent K^+^-transporters (*trkH*, *trkA*, *kup*) that may control the flux of potassium ions into the cells. As mentioned previously, potassium is important in pH homeostasis of acidophiles due to its role in generating a positive membrane potential and reducing the influx of protons when they are under pH stress. The accumulation of potassium ions along with its counter ion, glutamate, have also previously been described to form part of the primary response to osmotic stress in microorganisms (Sleator and Hill, 2002; Epstein, 2003). In extremely halotolerant bacteria, such as *Halorubum saccharovorum* and *H. trapanicum*, potassium has been shown to almost exclusively contribute to osmotic adjustment while in moderately tolerant species such as *H. mediterranei* and *H. gibbonsii*, it contributes to over 70% of osmotic adjustment (Shabala and Shabala, 2011). Therefore, it is hypothesized that these low affinity K^+^ uptake systems have an important role in salt stress tolerance through osmotic adjustment by members of the *Acidihalobacter* genus.

The genome of *A. prosperus* DSM 14174 was the only species in the genus to contain an operon consisting of genes for high affinity potassium transporting ATPase chains A, B and C (*kdpABC*), an osmosensitive K^+^ channel histidine kinase (*kdpD*) and the two- component system response regulator (*kdpE*). In addition to the low affinity K^+^ transport systems, it has previously been shown that osmotic stress and K^+^ limitation are responsible for strongly inducing the transcription of a *kdp* operon. The Kdp system was shown to play a role in increasing the osmotolerance of *Escherichia coli*, *Staphylococcus aureus* and *Sinorhizobium meliloti* by allowing scavenging of K^+^ when concentrations are low (Laimins et al., 1981; Gowrishankar, 1985; Domínguez-Ferreras et al., 2009; Price-Whelan et al., 2013). The genes *kdpA*, *kdpB*, and *kdpC* were found to be upregulated by 27-, 15-, and 8-fold, respectively, in *Cronobacter sakazakii* under osmotic stress (Lang et al., 2017). It is possible that the presence of this system in *A. prosperus* DSM 14174 renders this strain more resilient to salt stress than the other members of the genus when inhabiting K^+^ limited environments.

The synthesis of osmoregulated periplasmic glucans, which have been reviewed for Proteobacteria by Bohin (2000) may provide another well-known mechanism of osmotic adaptation in *Acidihalobacter* members (Kindzierski et al., 2017). In *E. coli*, *Halomonas elongata* and other Gram-negative bacteria, osmoregulated periplasmic glucans are synthesized at low osmolarity, suggesting that they play a role in the initial adaptation against osmotic stress (Kennedy, 1982; Miller et al., 1986; Kindzierski et al., 2017). We suggest a similar role for osmoregulated periplasmic glucans in *Acidihalobacter* members. Genes *opgH* and *opgG* coding for glucosyltransferase and glucan biosynthesis precursors, respectively, that are involved in the synthesis of these glucans were found on all *Acidihalobacter* genomes. *A. prosperus* DSM 5130^T^ also had genes for *opgD* which is another glucan biosynthesis precursor. It has previously been seen that the *opgD* and *opgG* genes can be either present or absent on certain genomes and that the interactions between *opgH* and *opgG*/*opgD* have evolved differently in different species (Kindzierski et al., 2017).

Sucrose has previously been identified as a non-accumulated disaccharide that provides osmoprotection in *S. meliloti* (Gouffi et al., 1999). In this species, sucrose does not accumulate as an osmolyte or a precursor of osmolytes but, rather, indirectly enhances the levels of glutamate and N-acetylglutaminylglutamine in order to help increase growth at high salt (Sleator and Hill, 2002). The gene for sucrose synthase (*sus*) was found in all members of the *A. prosperus* species. However, genes for sucrose phosphate synthase (*sps*) and sucrose phosphorylase (*spp*) were found on all the genomes, suggesting that both sucrose synthesis and metabolism occurs in all the members of the *Acidihalobacter* genus and that this may play a role in the initial response to osmotic stress, as has previously been described for *S. meliloti* (Gouffi et al., 1999).

### Osmoprotectants in *Acidihalobacter* spp.

The primary response to osmotic stress mentioned above is a temporary response, as potassium glutamate increases intracellular osmolarity, inducing a negative effect on cellular metabolism when salt stress is prolonged. Therefore, the effect of potassium glutamate is to act as a signal of osmotic stress and to stimulate the secondary response, i.e., the removal of potassium glutamate and the synthesis or uptake of osmoprotective compounds, known as osmoprotectants, osmolytes or compatible solutes (Sleator and Hill, 2002). Osmoprotectants are low molecular weight organic molecules that can accumulate to high concentrations in cells without affecting cellular processes due to their high solubility and their ability to not interact with proteins as a result of being uncharged at physiological pH. When cells are under stress, osmoprotectants help by stabilizing proteins and by balancing internal solute levels to match the immediate environment (Roeßler and Müller, 2001).

In a recent study, trehalose was identified as one of, or the sole, osmoprotectant/compatible solute in members of the salt sensitive, acidophilic, iron/sulfur oxidizing *Leptospirillum* and *Acidithiobacillus* spp., with the exception of *A. thiooxidans* (Galleguillos et al., 2018). Members of the *Acidihalobacter* spp. appear to be unique among the iron and sulfur oxidizing acidophiles in their absence of genes for the synthesis of trehalose. Potential osmoprotectants that can be used instead by the members of the *Acidihalobacter* genus are discussed below.

**FIGURE 5:**
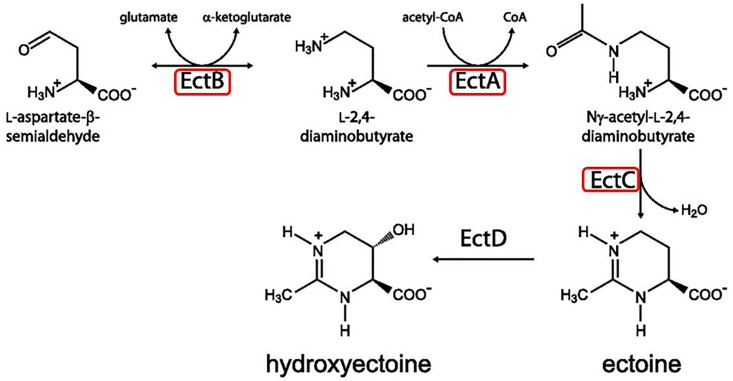
The ectoine biosynthesis pathway. *Acidihalobacter* genomes contain the genes *ectABC* (highlighted in red boxes) but not *ectD* and therefore are likely to synthesize ectoine but not hydroxyectoine through the pathway shown. Reproduced from Ofer et al. (2012) with permission from American Society for Microbiology.

Ectoine is one of the most abundant osmolytes in nature and was first described in the extremely halophilic sulfobacterium *Ectothiorhodospira halochloris* (Galinski et al., 1985). It is a zwitterionic molecule that has been found to improve protein folding. It also has been found to protect biomolecules from extremes environments, including solute, temperature and chemical stresses (Barth et al., 2000). Genes coding for the enzymes required for ectoine biosynthesis were found in each of the *Acidihalobacter* spp. genomes. The detection of these genes in the *Acidihalobacter* genomes suggested that ectoine contributed to the osmotolerance of the species in this genus (Ossandon et al., 2014; Khaleque et al., 2017b,c). This was confirmed in recent proteomic studies that showed increased abundance of ectoine transporters (50 fold increase) and ectoine synthase (422 fold increase) in DSM 5130^T^ and *A. prosperus* DSM 14174, respectively, when they were grown at high salt stress (Dopson et al., 2017; Khaleque et al., 2018b), thereby confirming that ectoine is an important osmoprotectant in these two strains. Ectoine biosynthesis in *Acidihalobacter* can proceed through a pathway involving the products of genes for diaminobutyrate-pyruvate aminotransferase (*ectA*), L-2,4-diaminobutyric acid acetyltransferase (*ectB*) and L-ectoine synthase (*ectC*) (Louis and Galinski, 1997; Figure 5). Genomes of members of the *Acidihalobacter* genus were found to contain genes for a *marR*-like regulatory protein associated with the ectoine operon that has previously been implicated in the transcriptional regulation of the ectoine operon in the halotolerant obligate methanotroph *Methylomicrobium alcaliphilum* strain 20Z in response to an increased salinity (Mustakhimov et al., 2010). Furthermore, transporters for ectoine encoded by genes *ehu*ABCD were also identified on all of the genomes as were the genes *eut*BCD, which have previously been indicated to have roles in ectoine catabolism as part of the *eut*ABCD operon (Jebbar et al., 2005). *Leptospirillum* spp. are the only other acidophilic species whose genomes contain genes for the biosynthesis and transport of this osmoprotectant (Mosier et al., 2013). *Leptospirillum* group II bacteria also contain the ectoine dioxygenase/hydroxylase (*ectD*) gene for the conversion of ectoine to hydroxyectoine, which has also been implicated as an osmoprotectant in this group of bacteria (Mosier et al., 2013; Figure 5). However, as the *Acidihalobacter* genomes do not contain the *ectD* gene it is not known if hydroxyectoine is synthesized by members of this genus as an osmoprotectant.

Previously proline was found to be the predominant compatible solute used by the moderate halophile *Halobacillus halophilus*, when faced with increasing osmotic stress (Saum and Müller, 2007). In the acidophilic sulfur oxidizing *A. caldus* SM-1, enzymes involved in the proline synthesis were only observed in cells grown at 0.5 M sodium chloride (Guo et al., 2014). Proline biosynthesis from glutamate proceeds through three enzymes in this microorganism: pyrroline-5-carboxylate reductase (*proH*), glutamate 5-kinase (*proJ*) and a glutamate semialdehyde dehydrogenase (*proA*) (Saum and Müller, 2008). The inspection of the genomes in this study revealed the presence of these three genes in all *Acidihalobacter* genomes. Furthermore, in *A. prosperus* DSM 5130^T^, *A. prosperus* DSM 14174 and “*A. ferrooxidans*” DSM 14175, the *proH* gene was part of an operon consisting of a proline synthase homolog (*yggS*), *proH*, an integral membrane protein involved in osmotic shock response (*yggT*) and a DUF167 domain protein (*yggU*). In “*A. ferrooxidans*” DSM 14175, the *yggS* gene was not found as part of the same operon but was present elsewhere on the genome.

While the synthesis of compatible solutes is of importance in microorganisms faced with salt stress, the transport of these compounds can also provide osmoprotection. For example, although the *Acidihalobacter* genomes did not contain genes for the synthesis of glycine betaine, it is still possible that they can use it as an osmoprotectant because the transport and uptake of proline and glycine betaine can occur through the same transporters (Waditee et al., 2002). All four *Acidihalobacter* genomes showed the presence of genes for the proline/glycine betaine ABC-transport-system permeases, *proV* and *proW*. However, only *A. prosperus* species carried glycine betaine binding protein genes *opuAC.* The opuC protein has previously also been implicated in the transport and accumulation of ectoine in *Bacillus subtilis* under osmotic stress (Jebbar et al., 1997). *A. prosperus* F5 additionally carried the glycine betaine ABC transport permease genes, *opuAB*. “*A. ferrooxidans*” DSM 14175 lacked both *opuAC* and *opuAB* but carried a gene for an alternative proline/glycine betaine binding ABC transporter protein *proX*.

Taurine appears to be another osmoprotectant that can be accumulated in certain members of the *Acidihalobacter* genus. Taurine has previously been found to be used as an osmoprotectant in microbial communities from biofilms at the Richmond mine, Iron Mountain, though none of the bacteria or archaea in the study were able to synthesize it (Mosier et al., 2013). The study found that taurine uptake proteins were produced by *Sulfobacillus* spp. and these were predicted to provide osmoprotection by providing a means of uptake and accumulation of this amino acid (Mosier et al., 2013). Similarly, the genes *tauABC* encoding proteins involved in taurine uptake have also been found on the genome of *A. prosperus* DSM 5130^T^, whereas “*A. ferrooxidans*” DSM 14175 only contained *tau*B and *A. prosperus* DSM 14174 and *A. prosperus* F5 genomes did not contain any *tau* genes. The genomes of both DSM 5130^T^ and “*A. ferrooxidans*” DSM 14175 lacked genes for the utilization of taurine (*tauD*), suggesting that in these species, taurine may accumulate as an osmoprotectant rather than for use as a metabolite.

### Mechanisms of Dealing With Chloride Ion Stress

**FIGURE 6:**
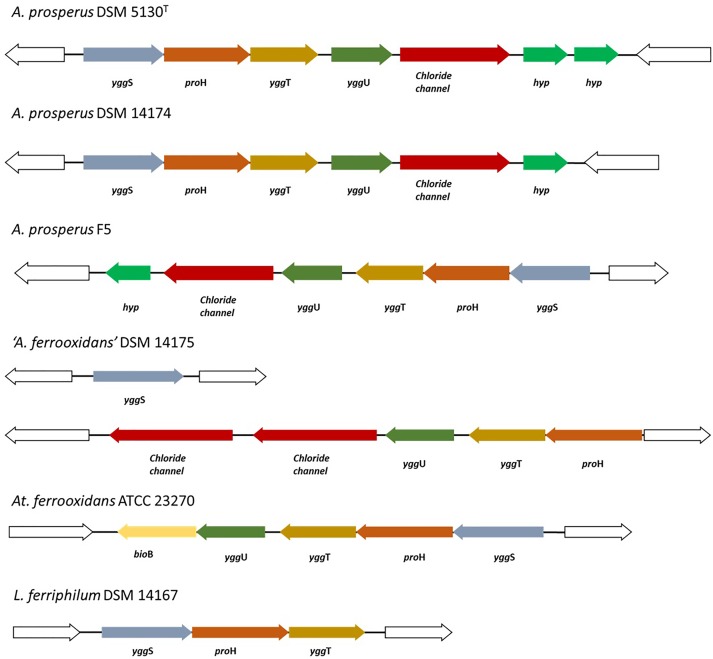
Comparison of the *yggS-proH-yggT-yggU* operon in members of the *Acidihalobacter* genus against that in salt sensitive, acidophilic *A. ferrooxidans* ATCC 23270 and the moderately salt tolerant *L. ferriphilum* DSM 14167. The members of the *Acidihalobacter* genus all encoded chloride ion channel proteins in the operon.

As mentioned previously, chloride ion stress is a limiting factor for growth of acidophilic bioleaching microorganisms. Therefore, the genomes of the *Acidihalobacter* spp. were searched for genes that may have a role in chloride ion tolerance. Multiple genes encoding chloride ion channel proteins were present on all the genomes of the *Acidihalobacter* species. Of special interest were the chloride ion channels found directly downstream of the previously mentioned *yggS-proH-yggT-yggU* operon in *A. prosperus* DSM 5130^T^, *A. prosperus* DSM 14174 and *A. prosperus* F5. The “*A. ferrooxidans*” DSM 14175 genome was missing the *yggS* gene in the operon but contained two separate genes encoding chloride ion channels. The genomes of the salt sensitive *A. ferrooxidans* ATCC 23270 and *L. ferriphilum* DSM 14167 were searched to identify whether this operon was present on their genomes. While the genome of *A. ferrooxidans* ATCC 23270 was found to contain an *ygg*S-*pro*H-*ygg*T-*ygg*U operon, chloride channels did not form part of this operon. Similarly, in *L. ferriphilum* DSM 14167, only *ygg*S-*pro*H-*ygg*T were present as part of the operon but the other genes were absent. It is hypothesized that the proteins of the *yggS-proH-yggT-yggU* operon in *Acidihalobacter* spp. may be co-expressed as a result of chloride ion entry to the cells and therefore may provide a mechanism of dealing with chloride stress in *Acidihalobacter* species. A comparison of the operons in the different microorganisms is shown in Figure 6.

Another protein that has recently been found to decrease in abundance by 2 fold in *A. prosperus* DSM 5130^T^ and 2.6 fold in *A. prosperus V6* at high salt is the outer membrane protein A, encoded by *omp*A (Dopson et al., 2017; Khaleque et al., 2018b). Previous studies have suggested that a decrease in these proteins results in a reduction of pores in the outer membrane that may inhibit chloride from entering the cells (Csonka and Hanson, 1991; Dopson et al., 2017; Khaleque et al., 2018b). Genes encoding ompA precursors were found on the genomes of all *Acidihalobacter* species, and we hypothesize they have similar roles in *A. prosperus* F5 and “*A. ferrooxidans*” DSM 14175. Furthermore, outer membrane protein A has been shown to have a role in maintaining the stability and integrity of the bacterial membrane and previous studies have suggested that the periplasmic domain of this protein has a role in both acid and osmotic stress tolerance in *E. coli* (Koebnik, 1995; Wang et al., 2016). This suggests that ompA may play an important role in the tolerance of the *Acidihalobacter* spp. to chloride, osmotic stress and acid stress.

### Astrobiological Significance

Extreme halophiles and the terrestrial environments they inhabit are being used as analogs for providing useful insights into the potential for life elsewhere in the universe (DasSarma, 2006; Cabrol, 2018). For example, it is widely accepted that Mars once had a liquid ocean that could potentially have supported life but it subsequently dried out leaving the arid surface of Mars today (Citron et al., 2018). However, as Mars dried out acidic, saline liquid waters were intermittently available and salts were precipitated from Martian brines (Tosca et al., 2008; Tosca et al., 2011). Analog hypersaline environments on Earth such as the Salares of Northern Chile, the Dead Sea, the Basque Lakes of British Colombia and many others are being exploited to determine what organisms are present in such environments and how they tolerate high concentrations of salts (Bodaker et al., 2010; Pontefract et al., 2017; Fox-Powell and Cockell, 2018). Within the next few years, missions will set out to search for biosignatures on Mars (Vago et al., 2017) and it is imperative to develop ideas and models of the sort of life, or evidence of past life, we should be looking for and in which Martian environments.

## Conclusion

Comparative genomics is the first step in identifying how microorganisms differ on a molecular level and helps to identify genes conserved among species as well as genes that may give an organism its unique characteristics. The genomic comparison of the members of the *Acidihalobacter* genus has helped to extend the knowledge of the differences in their mechanisms of tolerance to salt stress thereby determining their usefulness for saline water bioleaching processes. The basic features for osmotolerance for the members of the *Acidihalobacter* genus appear to be the ability to accumulate potassium and synthesize osmoregulated periplasmic glucans and sucrose as the primary response to salt stress and then to replace these with osmoprotectants as salt stress increases. Differences could be seen in the genes coding for proteins involved in halotolerance suggesting that the different members of the *Acidihalobacter* genus may use a different mechanisms for surviving high salt stress. Further proteomic work is required to confirm the preferential mechanisms of halotolerance used by the extremely acidophilic members of this genus.

## Supporting Information

The full list of gene annotations showing encoded proteins can be found at the following link: https://drive.google.com/open?id=0BwTiq6bJgkC_T1NHMGRuLUFjTEE.

## Author Contributions

HK, EW, CG, and DH conceived and designed the experiments. HK, CG, and RS performed the experiments and analyzed the data. EW and AK contributed to the materials and analysis tools. HK and EW wrote the manuscript. All authors read and approved the final manuscript.

## Conflict of Interest Statement

RS was employed by company Sodexo Australia. The remaining authors declare that the research was conducted in the absence of any commercial or financial relationships that could be construed as a potential conflict of interest.

## Footnotes

1 http://rast.nmpdr.org/

2 http://www.genome.jp/kegg/
